# *Toxoplasma gondii* IgG serointensity and cognitive function in bipolar disorder

**DOI:** 10.1186/s40345-024-00353-8

**Published:** 2024-08-23

**Authors:** Paul Rensch, Teodor T. Postolache, Nina Dalkner, Tatjana Stross, Niel Constantine, Aline Dagdag, Abhishek Wadhawan, Farooq Mohyuddin, Christopher A. Lowry, Joshua Joseph, Armin Birner, Frederike T. Fellendorf, Alexander Finner, Melanie Lenger, Alexander Maget, Annamaria Painold, Robert Queissner, Franziska Schmiedhofer, Stefan Smolle, Adelina Tmava-Berisha, Eva Z. Reininghaus

**Affiliations:** 1 Division of Psychiatry and Psychotherapeutic Medicine, Medical University of Graz, Graz, Austria; 2 Department of Psychiatry, University of Maryland School of Medicine, Baltimore, Maryland USA; 3 Center for Research on Aging, University of Maryland School of Medicine, Baltimore, Maryland USA; 4 Rocky Mountain Mental Illness Research Education and Clinical Center (MIRECC), Aurora, CO USA; 5 VISN 5 Capitol Health Care Network Mental Illness Research Education and Clinical Center (MIRECC), Baltimore, Maryland USA; 6 Institute of Human Virology, Department of Pathology, University of Maryland School of Medicine, Baltimore, Maryland USA; 7 Department of Psychiatry, Saint Elizabeths Hospital, Washington, DC USA; 8 Department of Integrative Physiology, University of Colorado Boulder, Boulder, CO USA; 9 Department of Physical Medicine and Rehabilitation, University of Colorado Anschutz Medical Campus, Aurora, CO USA

**Keywords:** Bipolar disorder, Toxoplasmosis, *Toxoplasma gondii*, Cognition

## Abstract

**Background:**

Alongside affective episodes, cognitive dysfunction is a core symptom of bipolar disorder. The intracellular parasite *T. gondii* has been positively associated with both, the diagnosis of bipolar disorder and poorer cognitive performance, across diagnostic boundaries. This study aims to investigate the association between *T. gondii* seropositivity, serointensity, and cognitive function in an euthymic sample of bipolar disorder.

**Methods:**

A total of 76 participants with bipolar disorder in remission were tested for *T. gondii*-specific IgG and IgM antibodies and for cognitive performance using neuropsychological test battery. Cognitive parameters were categorized into three cognitive domains (attention and processing speed, verbal memory, and executive function). Statistical analysis of associations between continuous indicators of cognitive function as dependent variables in relationship to *T. gondii*, included multivariate analyses of co-variance for seropositivity, and partial correlations with IgG serointensity in IgG seropositives. All analyses were controlled for age and premorbid IQ.

**Results:**

In seropositives (*n* = 27), verbal memory showed significant inverse partial correlations with IgG antibody levels (short delay free recall (*r=*–0.539, *p =* 0.005), long delay free recall (*r=*–0.423, *p =* 0.035), and immediate recall sum trial 1–5 (*r*=–0.399, *p* = 0.048)). Cognitive function did not differ between IgG seropositive and seronegative individuals in any of the cognitive domains (*F* (3,70) = 0.327, *p* = 0.806, *n* = 76). IgM positives (*n* = 7) were too few to be analyzed.

**Conclusions:**

This investigation is the first to show an association between *T. gondii* IgG serointensity and memory function in a well-diagnosed bipolar disorder sample. It adds to the existing literature on associations between latent *T. gondii* infection and cognition in bipolar disorder, while further research is needed to confirm and expand our findings, eliminate potential sources of bias, and establish cause-effect relationships.

## Background

Bipolar Disorder (BD) is a chronic psychiatric disorder causing recurring periods of depression and mania. BD has a lifetime prevalence of 1.02% (Moreira et al. 2017) and causes an average of 12.89 years of potential life lost in affected patients (Chan et al. 2022). Besides pathological mood swings, a core symptom of BD is cognitive dysfunction that even persists during euthymia (Bourne et al. 2013; Cullen et al. 2016). Studies have shown that cognitive deficits predict decrements in psychosocial and occupational functioning (Gilbert and Marwaha 2013), and reduced quality of life (Mackala et al. 2014).

*Toxoplasma gondii (T. gondii)* is a protozoan intracellular parasite with a worldwide distribution (Pappas et al. 2009) that infects up to a third of the world’s population (Montoya et al. 2004). While it infects all warm-blooded animals, the parasite can only sexually reproduce in cats. The parasite infection can be divided into three distinct stages: sporozoites - within sporulated oocysts, tachyzoites that rapidly invade and multiply, and bradyzoites that replicate slowly within tissue cysts. In approximately half of the cases of toxoplasmosis, humans become infected through a foodborne route consisting of ingestion of *T. gondii* oocysts shed in the feces of infected cats and contaminating food during food preparation, or tissue cysts from the meat of infected animals.

While *T. gondii* is especially known for its possible negative effects on fetal development (Ahmed et al. 2020), infection acquired postnatally on the other hand is usually asymptomatic (“latent toxoplasmosis”) in immunocompetent individuals and rarely needs treatment (Montoya et al. 2004). Nevertheless, once an organism is infected with *T. gondii*, the clearance of the microorganism is virtually impossible. Most adult infections therefore are chronic, with only minimal reactivations of the pathogen (Dubey 2021).

Diagnosis of infection is commonly made with serological methods detecting *T. gondii*-specific antibodies. Acute infection is accompanied by elevated IgM, which can be detected as soon as one week after infection. It peaks after 1–3 months and usually disappears over the following months; yet, in some patients IgM can also be observed for many years after the acute infection (Reiter-Owona 2005; Liesenfeld et al. 1997). Subsequently, IgG appears around two weeks after infection and persists for life in immunocompetent hosts (Nichols and Jones 2017).

Despite *T. gondii* being considered harmless in immunocompetent individuals for a long time, recent investigations highlight that the parasite might also be linked to a wide array of neuropsychiatric disorders (Milne et al. 2020; Virus et al. 2021; Sutterland et al. 2015). There is evidence of associations between the parasite and epilepsy (Ngoungou et al. 2015), schizophrenia (Torrey et al. 2007), as well as suicidal behavior, across diagnostic categories (Arling et al. 2009; Okusaga et al. 2011; Pedersen et al. 2012; Postolache et al. 2021). Furthermore, possible links to obsessive-compulsive disorder (Sutterland et al. 2015) and Alzheimer’s disease (Bayani et al. 2019; Nayeri Chegeni et al. 2019) have been reported. In contrast, studies examining associations with depression have shown heterogeneous and predominantly negative results (Gale et al. 2021; Liu et al. 2022).

The association between *T. gondii* and BD has been reviewed by several meta-analyses identifying significant relationships in all ages (Sutterland et al. 2015; Barros et al. 2017), as well as in a subpopulation under 40 years of age (Snijders et al. 2019). A recent publication with 12,690 participants (4,021 with BD and 8,669 controls) supported these findings and reported that persons with BD had significantly greater odds of seropositivity for *T. gondii* than controls (Cossu et al. 2022).

Although cognitive function is an important parameter of outcome in BD, to our knowledge only two studies have examined a link between *T. gondii* and cognitive performance, but with inconsistent results. Dickerson et al. (*n* = 347) found significant associations between *T. gondii* IgM, but not IgG serointensity, and certain cognitive parameters (overall cognitive function, delayed memory and visuospatial/constructional), but not for immediate memory, language, or attention. Moreover, IgG serointensity and cognition scores showed no association in their study (Dickerson et al. 2014). Tanaka et al. (*n* = 32) found no association between any antibody levels and overall neurocognitive function (Tanaka et al. 2017).

Interestingly, in healthy individuals, a meta-analysis by de Haan et al. with 13,289 participants reported an association between *T. gondii* seropositivity and mild cognitive impairment. In this analysis, individuals with latent *T. gondii* infections had inferior performance in four cognitive domains: processing speed, working memory, short-term verbal memory, and executive function, relative to seronegative individuals (Haan et al. 2021).

In summary, prior studies reported inconsistent results on associations between latent *T. gondii* infections and cognitive dysfunction in BD, while one meta-analysis in healthy individuals was able to identify associations of *T. gondii* seropositivity with distinct cognitive domains. Therefore, the current investigation aimed to investigate the association between *T. gondii* seropositivity, serointensity, and cognitive domains in a well-diagnosed BD population. We hypothesized that in euthymic bipolar patients, the *T. gondii*-seropositive individuals would manifest more severe cognitive impairments relative to their seronegative individuals. In addition, we assumed that there would be negative associations between serointensity and measures of cognitive performance in *T. gondii* seropositives.

## Methods

### Sample description and inclusion criteria

We measured *T. gondii*-specific antibodies in a total of 126 individuals with BD as part of the ongoing “BIPLONG” study project at a dedicated BD outpatient clinic at the Medical University of Graz, Department of Psychiatry and Psychotherapeutic Medicine. BIPLONG aims to assess psychiatric symptoms and history in association with metabolic, lifestyle, genetic, biological, brain imaging, and cognitive parameters in individuals with BD in a longitudinal design. After baseline measurement, there are follow-up measurements every six months. In the current investigation, we used data from the baseline measurement. For more details about the BIPLONG project, please see earlier publications by our study group (Reininghaus et al. 2014; Dalkner et al. 2021; Fellendorf et al. 2021).

Participants were diagnosed with BD according to the Structured Clinical Interview for DSM IV (SCID-I) by a trained psychiatrist or psychologist with the German Version (Wittchen et al. 1997). Furthermore, participants had to be euthymic at the time of testing and had to have a complete cognition data set. Euthymia was determined by a score of ≤ 12 on the Hamilton Depression Rating Scale (HAMD) (Hamilton and Guy 1976) and a score of ≤ 8 on the Young Mania Rating Scale (YMRS) (Young et al. 1978). Exclusion criteria were a premorbid IQ of < 80 tested with the multiple choice vocabulary test (MWT-B) (Lehrl 2005) and an age of ≥ 70 years.

Out of the 126 individuals considered, 38 participants did not meet the criteria for euthymia; three were over 70 years of age, and nine participants had to be excluded because of missing cognition data. In total, we were able to include 76 participants in this investigation.

Additionally, for computing correlations between serointensity and cognitive parameters, we used only the serointensity higher than positivity thresholds. Therefore, 49 patients who tested seronegative for *T. gondii* were excluded and, thus, the sample comprised of 27 participants for the serointensity component of the analysis.

### Serological analysis

For each participant, a blood sample was obtained on the day of cognitive testing. Plasma was isolated from blood samples, stored, and shipped on dry ice until testing. The serological analysis was conducted by the Institute of Human Virology and Department of Pathology at the University of Maryland School of Medicine, Baltimore, USA. *T. gondii* antibodies, specific for IgG or IgM, were detected in subjects using commercial ELISA methods (IBL International, Hamburg, Germany). Briefly, diluted plasma was added to antigen-coated plates, and the specific antibodies were detected using an anti-IgG or anti-IgM peroxidase-labeled conjugate, followed by the addition of a TMB substrate. Color development was detected at 450 nm. Intensity was measured and the concentration of antibodies was determined as an Antibody Index based on the OD of a calibrator, a Calibrator Factor, and the sample OD. Sample results with an Antibody Index of greater than 1.1 were considered seropositive for IgG- or IgM-specific antibodies (as per the manufacturer’s guidelines).

### Neuropsychological assessment

We used a neuropsychological test battery including:

- *Attention and processing speed*: Trail Making Test part A (TMT-A) (Reitan 1956), the word- and color-naming trials from the Color and Word Interference Test by J. R. Stroop (Bäumler and Stroop 1985), the d2 Test of Attention Revised (d2-R) (Brickenkamp et al. 2010), and the digit-symbol-coding test from the “Hamburg-Wechsler-Intelligenztest für Erwachsene” (HAWIE-R) (Hamburg-Wechsler 1994). The latter is a German version of the “Wechsler Adult Intelligence Scale” (WAIS) (Wechsler 1955).
- *Verbal memory*: German Version of the California Verbal Learning Test (CVLT) with the subscales “immediate recall sum trial 1–5”, “short delay free recall”, “short delay cued recall”, “long delay free recall” and “long delay cued recall”(Niemann et al. 2009); and the digit span forward test from the WAIS.
- *Executive function*: Trail Making Test Part B (TMT-B), digit span backwards test from the WAIS, and the interference trial from the Color and Word Interference Test by J. R. Stroop.

Individual parameters were categorized into a cognitive domain according to the tests’ manuals. Since we lack a specific working memory domain, we incorporated the Digit Span Forward data into the memory domain, as it also measures verbal short-term memory, and the Digit Span Backwards data into executive function, as backward span is a more sensitive measure of working memory than the forward span condition (Hamburg-Wechsler 1994; Wechsler 1955).

### Descriptive analyses and sample characteristics

Descriptive analyses were conducted using independent sample t-tests and chi-square tests. The normal distribution of the variables were checked visually using histograms and the Kolmogorov-Smirnoff and Shapiro-Wilk tests, respectively. In cases where the normal distribution was not assumed, nonparametric Mann-Whitney U tests were applied.

A total of 76 patients were included in this study, of which 43 (56.6%) were male and 33 (43.4%) were female. The median age was 43.1 years (*SD* 13.1) with an age range from 17.8 to 67.2 years. The median illness duration was 18.9 years (*SD* 12.4). Seven (9.2%) participants were seropositive for IgM antibodies and 27 (35.5%) were seropositive for IgG antibodies.

### Statistical analyses

Due to the small sample size of the participants who were seropositive for IgM (*n* = 7), we were not able to perform any statistical analyses in this sub-group because of limited power and substantial risk of type II error.

For IgG antibody levels a natural logarithm transformation was performed to correct for the left-skewed distributions. Raw cognitive test scores were converted into *z*-scores. For higher scores to represent higher performance, variables initially oriented negatively were reversed.

Neuropsychological tests used in the BIPLONG study project were categorized and summed up into three cognitive domain scores: (a) *attention and processing speed* (TMT-A, Stroop word- and color naming trials and d2-R); (b) *verbal memory* (CVLT subscales and digit span forward test); and (c) *executive function* (TMT-B, digit span backwards test and Stroop interference). These domain scores were again transformed into *z*-scores and functioned as primary cognitive outcome variables.

To compare differences in cognitive function between seropositive and seronegative patients, two-way multivariate analyses of co-variance controlling for age and IQ (MANCOVA) were computed with IgG seropositivity as the independent variable, and cognitive domain scores (attention and processing speed, verbal memory, executive function) as dependent variables.

To evaluate for associations between IgG antibody serointensity and cognitive domain scores in a subsample of seropositive participants only, we used partial correlations controlling for age and premorbid IQ. Seronegative participants were excluded from this part of the analysis, since serointensity is only biologically meaningful in seropositive patients.

All statistical analyses were computed using IBM SPSS version 27.0.

## Results

### Differences in cognitive domain scores between seropositive and seronegative groups

MANCOVA analysis to test for differences in cognitive domain scores (attention and processing speed, verbal memory, executive function) between IgG seropositive and seronegative participants, controlling for age and premorbid IQ, showed no significant multivariate main effect for IgG seropositive relative to IgG seronegative individuals (*F* (3,70) = 0.327, *p* = 0.806, *n* = 76); results are shown in Table 1.

**Table 1:** Demographic and clinical characteristics and cognitive domain scores among IgG seropositive and seronegative groups (*n* = 76)

		Seropositive for IgG	Seronegative for IgG	Statistical Test, *p* value
		*n* = 27 (35.5%)	*n* = 49 (64.5%)	
*Demographics*				
Age (years), mean *± SD*		47.16 (12.682)	40.92 (12.869)	***t*(54.39)=–2.041**, ***p*** **= 0.046**
Sex, *n* (%)				*Χ*^*2*^(1) = 1.735, *p* = 0.188
Male		18 (41.9%)	25 (58.1%)	
Female		9 (27.3%)	24 (72.7%)	
High School Graduates, *n* (%)		15 (55.6%)	33 (67.3%)	
Employed, *n* (%)		9 (33.3%)	14 (28.6%)	
*Clinical Characteristics*				
Illness Duration (years), mean *± SD*		22.93 (13.032)	16.71 (11.589)	***U*** **= 476.5**, ***z* = –2.009**, ***p*** **= 0.044**
Number of Affective Episodes				
Mania, mean *± SD*		5.40 (7.25)	6.98 (6.94)	*t*(66)= − 0.899, *p* = 0.377
Depression, mean *± SD*		9.74 (8.77)	9.47 (8.40)	*t*(66) = 0.125, *p* = 0.901
HAMD, mean *± SD*		4.67 (3.497)	5.12 (3.626)	*U* = 615.0, *z* = –0.508, *p* = 0.612
YMRS, mean *± SD*		0.67 (1.617)	1.33 (2.267)	*U* = 567.0, *z* = –1.262, *p* = 0.208
BD Type, *n* (%)				*Χ*^*2*^(1) = 0.910, *p* = 0.340
Type 1		17 (32.1%)	36 (67.9%)	
Type 2		10 (43.5%)	13 (56.5%)	
Cognitive Domain Scores from MANCOVA*				
Main Effect				*F*(3,70) = 0.327, *p* = 0.806
Attention and Processing Speed, mean *± SD*		–8.435 (3.422)	0.465 (3.356)	*F*(1) = 0.696, *p* = 0.407
Verbal Memory, mean *± SD*		–0.653 (4.736)	0.360 (4.892)	*F*(1) = 0.003, *p* = 0.955
Executive Function, mean *± SD*		–0.291 (2.233)	0.160 (2.113)	*F*(1) = 0.015, *p* = 0.904

Note: Significant values are indicated in bold (*p* < 0.05); *Differences in cognitive domain scores, results corrected for age and IQ; IgG = Immunoglobulin G specific for *T. gondii*; HAMD = Hamilton Depression Rating Scale, YMRS = Young Mania Rating Scale, BD = Bipolar Disorder

Table 1 also presents further demographic and clinical characteristics and compares variables between IgG seropositive and seronegative groups.

### Associations between *T. gondii* IgG serointensity in seropositives and cognitive domain scores

Partial correlation coefficients were computed to assess linear relationships between IgG serointensity and cognitive domain scores controlling for age and premorbid IQ in a subsample of seropositive patients (*n* = 27). There were no significant correlations between IgG serointensity and both “attention and processing speed” (*r=*–0.095, *p =* 0.651) and “executive function” (*r =* 0.115, *p =* 0.586). There was a tendency towards a negative correlation (*r=*–0.396, *p =* 0.050) between IgG serointensity and “verbal memory”. Based on this tendency further correlations for individual parameters in the verbal memory domain were explored.

Specifically, additional partial correlations in the subsample of seropositive patients between IgG antibody levels and the individual test scores in the verbal memory domain using the California Verbal Learning Test were done using Bonferroni corrected alpha levels of *p* = 0.008. Results showed a negative correlation between IgG serointensity and short delay free recall (*r=*–0.539, *p =* 0.005), long delay free recall (*r=*–0.423, *p =* 0.035), and immediate recall sum trial 1–5 (*r*=–0.399, *p* = 0.048). No significant correlations were found between IgG serointensity, and short delay cued recall (*r=*–0.364, *p* = 0.074) and long delay cued recall (*r=*–0.215, *p =* 0.302). After Bonferroni correction for multiple comparisons, only negative correlation with short delay free recall remained significant (*p* < 0.008). Coefficients and statistics of partial correlation analyses are presented in Table 2.

**Table 2:** Partial correlation results between IgG serointensity (in IgG seropositives) and cognitive parameters (*n* = 27)

	*r*	*p* value
*Cognitive Domain Scores*		
Attention and Processing Speed	–0.095	0.651
Executive Function	0.115	0.586
Verbal Memory	–0.396	0.050
*Individual Parameters in the verbal memory domain*		
CVLT immediate recall sum trial 1–5	–0.399	0.048*
CVLT Short Delay Free Recall	–0.539	0.005*
CVLT Short Delay Cued Recall	–0.364	0.074
CVLT Long Delay Free Recall	–0.423	0.035*
CVLT Long Delay Cued Recall	–0.215	0.302
WAIS Digit Span Forward Test	0.115	0.583

Note: All results are controlled for age and IQ; CVLT = California Verbal Learning Test, WAIS = Wechsler Adult Intelligence Scale

*Significant values, values, which remained significant after Bonferroni correction are indicated in bold

## Discussion

### Key findings

Our investigation aimed to determine associations between *T. gondii*-specific antibodies and cognitive function in a well-diagnosed BD sample. While domain scores for “attention and processing speed” and “executive function” showed no partial correlations with IgG serointensity, we found inverse associations between *T. gondii* IgG serointensity and all individual free recall parameters within the “verbal memory” domain; however, only short delayed free recall remained significant after Bonferroni correction. The free recall condition of CVLT assesses several memory processes such as encoding, learning, storage, and retrieval. Thus, our results suggest that the higher *T. gondii* IgG serointensity were, the lower the individual’s short-term verbal learning abilities. Interestingly, our results are also consistent with longitudinal studies that have previously reported that deterioration in many cognitive tasks in BD is often the result of confounding, except for memory recall (Malhi et al. 2007).

We did not find differences between groups seropositive and seronegative for *T. gondii* IgG in any cognitive domain assessed (attention and processing speed, verbal memory, and executive function). This coincides with previous studies in BD samples (Dickerson et al. 2014; Tanaka et al. 2017).

A small sample size of participants seropositive for IgM (*n* = 7) precluded us from attempting to reevaluate previously reported positive association between *T. gondii* IgM serointensity and certain measures of cognitive dysfunction (Dickerson et al. 2014), which later was not replicated (Tanaka et al. 2017).

Furthermore, seropositive individuals were on average older than seronegative individuals. Once infected, the host cannot eradicate *T. gondii* due to its ability to hide in intracellular cysts within neurons and glial cells, and to manipulate the immune system. Therefore, prevalence of chronic toxoplasmosis is known to increase with age (Wilking et al. 2016), and thus we accounted for age by statistical adjustment. We observed no other group differences between seropositive and seronegative groups in any demographic or clinical parameters that might be capable of confounding our results.

### Clinical implications

Our results indicate that *T. gondii* might be moderately associated with poorer performance in certain free recall subtasks in the verbal memory domain. On a clinical level, the parasite cannot yet be established as a risk factor for cognitive decline, solely based on the results from the present cross-sectional study. Consequently, this study should be interpreted as an addition to the existing literature on *T. gondii* infection, while being the first one to our knowledge showing significant partial correlations between *T. gondii* IgG and verbal memory parameters.

If cause and effect relations can be established in the future, *T. gondii* might, due to its worldwide distribution and high prevalence, still impose an additional risk to BD populations, which are already affected by cognitive deficits due to their underlying illness (Bourne et al. 2013). Higher *T. gondii* prevalence in BD has been shown in a number of meta-analyses (Sutterland et al. 2015; Barros et al. 2017; Snijders et al. 2019; Cossu et al. 2022), and therefore, makes BD patients even more susceptible to possible negative effects caused by the parasite. Since cognitive impairment also predicts psychosocial functioning and quality of life (Gilbert and Marwaha 2013; Mackala et al. 2014), identification of *T. gondii* as a risk factor and prognostic marker for cognitive outcome might lead to novel treatment approaches for BD patients. Especially, since a few psychotropics in the current clinical arsenal in BD have sizeable anti-*T. gondii* activity, consideration of anti-toxoplasma effects of certain psychotropic medications already used in BD would be necessary. For example, valproic acid has a considerable antiproliferative effect against *T. gondii* tachyzoites in vitro (Jones-Brando et al. 2003) relative to lithium or lamotrigine (Fond et al. 2014), resulting in reductions in tissue cyst load in glial nodules and perivascular infiltration of lymphocytes (Enshaeieh et al. 2021), and thus, could become, after confirming in randomized clinical trials, a mood stabilizer of choice in BD patients with high *T. gondii* serointensity.

### Possible cellular and molecular mechanisms

The potential neurobiological mechanisms and mediators the parasite uses to specifically alter cognition include cellular effects (apoptosis, deafferentation, changes in brain volume in different brain areas), changes in neurotransmitters – catecholamines and serotonin either by direct invasive effect of the parasite or indirectly, and via activation of the immune system. Additionally, proinflammatory cytokines may induce catabolism of tryptophan toward kynurenine pathways and production of neuroactive compounds, such as quinolinic acid, which may worsen cognition through brain tissue damage secondary to increased excitotoxicity, and the neuroprotective product kynurenic acid, which may be directly involved in expression of cognitive deficits (Miller et al. 2009).

### Directions for future research

In future studies larger sample sizes or meta-analyses will be necessary to provide stronger evidence for associations reported in our study. Moreover, longitudinal designs will be necessary to confirm if *T. gondii* serointensity in seropositives can predict cognitive decline, function as a catalyst, or even be, at least in part, a causal factor of cognitive decline in a subgroup of BD patients. It might also be worthwhile to investigate the memory component in more detail and to use tests for figural recall and working memory in addition to verbal memory.

Identification and targeting of the molecular and cellular mechanisms mediating the link between *T. gondii* and cognitive deficits may lead to novel targets and treatment modalities to be tested in their own right.

Furthermore, there is a need in future studies to use broader serological testing to identify specific serotypes of *T. gondii*, the source of infection via more virulent oocysts versus tissue cysts, as well as co-occurring, and reciprocally potentiating, infections with other chronic latent pathogens. While evidence is emerging that some serotypes of *T. gondii* may confer worse clinically relevant outcomes (Evangelista et al. 2023), it is possible that certain strains (Brito et al. 2023) or modes of infection (e.g., tissue cyst versus oocyst) of *T. gondii*, under some conditions, serve as microbial “Old Friends”— promoting immunoregulation (Tenorio et al. 2011; Wagner et al. 2009), and thus, reducing chronic low-grade immune activation, which has been associated with BD (Jones et al. 2021).

### Strengths and limitations

Strengths of this study were its homogeneous and well-diagnosed BD (according to SCID-I) study population, and a clearly defined euthymic state at the time of testing to avoid cognitive alterations caused by mood state episodes. Euthymia was a mandatory inclusion criterion, securing that the mood-state dependent cognitive deficits are not the driver of our associations.

This study cannot provide evidence for cause-and-effect inferences due to its observational stance and cross-sectional design. Another limiting factor was the small sample size in the evaluation of associations with IgM seropositivity and serointensity (*n* = 7) that precluded us from doing analyses in this sub-group. As IgM antibodies can persist years after infection, and could be elevated with reinfection with a different strain, and because the IgM can give false positive results and thus have a limited use in determining incidence, severity and timing of *T. gondii* infection (Dhakal et al. 2015), we have not integrated analysis of IgM seropositivity and serointensity into our analysis of IgG serointensity.

Furthermore, we did not have a healthy control comparison. Therefore, we cannot estimate if shown cognitive effects differ in individuals with BD (having multiple and potentially interactive liabilities like the recurrent affective disorder, the treatment effects, and finally the *T. gondii* infection) than in those reported in healthy controls (Haan et al. 2021). We also did not analyze effects of medications, and thus, there is a possibility that medications commonly used in BD and known to impact both cognitive function and the risk of dementia, either inducing worsening or improvement (Leopold and Quante 2023; Pigoni et al. 2020; Wingo et al. 2009; Forlenza et al. 2011), could have been differentially represented in the *T. gondii* positive versus negative groups, and thus either leading to spurious results or masking true associations. We also did not analyze a potential confounding, moderating or mediating role of body mass index (BMI) and metabolic factors such as dyslipidemia and diabetes type II, relevant due to the positive associations between obesity and metabolic dysregulation with both cognitive dysfunction in BD (Lackner et al. 2016; Ringin et al. 2023) and *T. gondii* infection (Reeves et al. 2013; Majidiani et al. 2016; Xu et al. 2020). Furthermore, we did not analyze possible interacting effects of alcohol or drug abuse, as well as socioeconomical factors. The latter could represent *T. gondii* infection risk factors and also could represent factors previously associated with a poor cognitive status and increased risk of dementia (Markon et al. 2020). We also acknowledge not measuring markers of distinct serotype and especially markers of the oocyst infection that appear to be more virulent, more neurotropic, with more tissue damage and inflammation (Dubey et al. 1981; Dubey 2006), and thus, potentially affecting cognition more strongly than the tissue cyst infection path would.

In addition, in the absence of a longitudinal study, it is not possible to exclude an alternative explanation for the reported associations. A reverse causality would implicate BD, in particular with preexisting, developmental, preprodromal or post-onset cognitive deficits, as a risk factor for infection and a more extensive and virulent *T. gondii* infection, perhaps by elevating risk factors for acquiring toxoplasmosis.

## Conclusions

This investigation is the first to show an association between *T. gondii* IgG serointensity and specific cognitive deficits in a subsample of BD patients (*n* = 27) seropositive for the parasite. Specifically, verbal memory parameters, and in particular, short delay free recall, were negatively correlated with IgG serointensity. This study adds to the existing literature on associations between latent *T. gondii* infection and cognition in BD, while further research is needed to confirm and expand our findings, eliminate potential sources of bias, and establish cause-effect relationships.

## Data Availability

Data are provided by the corresponding author on request.

## Abbreviations

BD: Bipolar Disorder
CVLT: California Verbal Learning Test
d2-R d2: Test of Attention Revised
ELISA: Enzyme-linked Immunosorbent Assay
HAMD: Hamilton Depression Rating Scale
HAWIE: Hamburg-Wechsler-Intelligenztest für Erwachsene
IgG: Immunoglobulin G
IgM: Immunoglobulin M
IQ: Intelligence Quotient
MANCOVA: Multivariate Analyses of Co-Variance
MWT-B: Multiple Choice Vocabulary Test
OD: Optical Density
SCID-I: Structured Clinical Interview for DSM IV
TMT-A: Trail Making Test Part A
TMT-B: Trail Making Test Part B
*T. gondii*: *Toxoplasma* *gondii*
WAIS: Wechsler Adult Intelligence Scale
YRMS: Young Mania Rating Scale
